# A large population-based association study between HLA and KIR genotypes and measles vaccine antibody responses

**DOI:** 10.1371/journal.pone.0171261

**Published:** 2017-02-03

**Authors:** Inna G. Ovsyannikova, Daniel J. Schaid, Beth R. Larrabee, Iana H. Haralambieva, Richard B. Kennedy, Gregory A. Poland

**Affiliations:** 1 Mayo Clinic Vaccine Research Group, Mayo Clinic, Rochester, MN, United States of America; 2 Department of Health Sciences Research, Mayo Clinic, Rochester, MN, United States of America; Istanbul University, TURKEY

## Abstract

Human antibody response to measles vaccine is highly variable in the population. Host genes contribute to inter-individual antibody response variation. The killer cell immunoglobulin-like receptors (KIR) are recognized to interact with HLA molecules and possibly influence humoral immune response to viral antigens. To expand on and improve our previous work with HLA genes, and to explore the genetic contribution of KIR genes to the inter-individual variability in measles vaccine-induced antibody responses, we performed a large population-based study in 2,506 healthy immunized subjects (ages 11 to 41 years) to identify HLA and KIR associations with measles vaccine-induced neutralizing antibodies. After correcting for the large number of statistical tests of allele effects on measles-specific neutralizing antibody titers, no statistically significant associations were found for either HLA or KIR loci. However, suggestive associations worthy of follow-up in other cohorts include B*57:01, DQB1*06:02, and DRB1*15:05 alleles. Specifically, the B*57:01 allele (1,040 mIU/mL; p = 0.0002) was suggestive of an association with lower measles antibody titer. In contrast, the DQB1*06:02 (1,349 mIU/mL; p = 0.0004) and DRB1*15:05 (2,547 mIU/mL; p = 0.0004) alleles were suggestive of an association with higher measles antibodies. Notably, the associations with KIR genotypes were strongly nonsignificant, suggesting that KIR loci in terms of copy number and haplotypes are not likely to play a major role in antibody response to measles vaccination. These findings refine our knowledge of the role of HLA and KIR alleles in measles vaccine-induced immunity.

## Introduction

Host genetic factors are believed to be responsible for up to 90% of measles vaccine-induced inter-individual antibody response variations [1]. Among these genetic factors, the Human Leukocyte Antigen (HLA) genes (on chromosome 6p21) have been a focus of interest since these highly polymorphic HLA genes play an important role in the regulation of immune response, including immunity to measles virus [2]. The main role of HLA class I and class II molecules is to present antigens to CD8+ and CD4+ T cells, respectively, thus initiating adaptive immune responses [3]. There is a large body of evidence demonstrating that immune responses to measles vaccine are in part, guided by polymorphisms of the HLA genes [4–8]. In this regard, several HLA class I (B*57:01, B*35:03) and class II (DQB1*06:02, DQB1*03:03, DRB1*07:01, DRB1*15:01) alleles have been consistently associated with variations in measles virus-specific antibody responses following measles vaccination [8].

While HLA alleles are highly polymorphic and difficult to fully investigate due to scarcity of some alleles, the functional consequences of the genes are less diverse. It is known that the type of peptides that HLA can bind—and the effectiveness of those bonds—is influenced by the shape of the binding region and the amino acids present in these regions that act to anchor peptides. This information allows us to view HLA in terms of downstream biological consequences and to re-categorize HLA alleles into “supertypes” based on their affinity to bind certain peptides. This simplified and practical view of the data may be more powerful and may offer more realistic information than the information gained by attempting to comprehend the impact of each individual allele [9].

Immune responses to measles are also influenced by various immunoregulatory genes (TLR, TRIM, CD46, SLAM, CD209, and others) [10–16]. Among these genes are the highly polymorphic immunoglobulin-like receptors (KIRs) expressed on the surface of natural killer (NK) cells [17]. A family of ~15 inhibitory and activating KIR polymorphic genes (located on chromosome 19q13.4) are expressed on human NK cells, which are important for antiviral innate and adaptive immune responses [18]. Unlike cytotoxic T cells, NK cells do not recognize specific pathogens, but instead express inhibitory KIR receptors that recognize polymorphic HLA class I ligands [19, 20]. NK receptors of the KIR family are known to interact with specific motifs on classical HLA class I (A, B, and C) molecules, and possibly influence immune response outcomes [21, 22]. To date, the involvement of the KIR genes in measles vaccine-induced immunity is unknown.

Several of our studies have demonstrated potential associations between HLA alleles and variations in measles vaccine-induced antibody responses [4, 5, 8]. Given the significance of the KIR genes for viral immunity/infection, we examined data previously obtained from three separate cohorts and examined whether variation within the KIR genes influences measles vaccine-induced antibody responses. Our goal was to examine HLA associations with humoral immune response outcome (neutralizing antibody titer) in a large population-based combined cohort of measles-mumps-rubella (MMR)-immunized subjects and to determine if KIR genotypes are associated with measles vaccine-induced antibodies.

## Materials and methods

### Study participants

The study population and recruitment methods described herein are similar or identical to those published for our previous studies [8, 10, 13, 23, 24].

Subjects from previously described cohorts were used for this study [10, 23, 24]. The combined study cohort was a large population-based sample of 3,191 healthy children, older adolescents, and adults (age 11 to 41 years) consisting of three independent cohorts: a Rochester cohort (n = 1,062); a San Diego cohort (n = 1,071); and a U.S. cohort (n = 1,058). The recruitment efforts, demographic and clinical characteristics of these cohorts have been previously published [10, 23, 24]. Specifically, 1,062 healthy children and young adults, ranging in age from 11 to 22 years, were recruited from Rochester, MN, between 2001–2009 (Rochester cohort), as previously published [8, 13]. Parental consent was obtained for all participants and each subject had written records of having received two doses of measles-mumps-rubella (MMR, Merck) vaccine. Of the 1,062 subjects for this study, 935 (88%) were successfully genotyped, assayed for immune response outcomes, and determined to have European ancestry (Table 1). The San Diego cohort consisted of 1,071 healthy older adolescents and healthy adults (age 18 to 40 years) enrolled by the Naval Health Research Center (NHRC) between 2005–2006, from armed forces personnel in San Diego, CA, as previously published [23, 25]. After excluding subjects without immune response outcome data and creating a subset of subjects with European ancestry, 695 (65%) subjects (San Diego cohort) remained for analysis. The U.S. cohort consisted of 1,058 healthy adults from the U.S. military (age 19 to 41 years) enrolled between 2010–2011 [24]. These subjects from the U.S. military represent a cross section of the U.S. population with proven MMR vaccine-induced immunity. Of the 1,058 individuals (US cohort), 876 (83%) subjects were successfully genotyped, assayed for immune response outcomes, and determined to have European ancestry using the STRUCTURE software [26]. The Institutional Review Boards of the Mayo Clinic (Rochester, MN) and the Naval Health Research Center (San Diego, CA) approved the study; written informed consent was obtained from each subject, from the parents of all children who participated in the study, as well as written assent from age-appropriate participants.

**Table 1:** Demographic and immune characteristics of the study subjects.

	Rochester (N = 935)	San Diego (N = 695)	US (N = 876)	Total (N = 2,506)
**Neutralizing antibody titer (mIU/mL)**^**a**^				
**Mean (SD**^b^**)**	1,313 (1,263)	1,257 (1,486)	1,237 (2,088)	1,271 (1,652)
**Q1, Q3**	435; 1751	372; 1526	334; 1404	383; 1607
**Median**	902	770	702	803
**Sex**				
**Female**	424 (45.3%)	201 (28.9%)	74 (8.45%)	699 (27.9%)
**Male**	511 (54.7%)	494 (71.1%)	802 (91.6%)	1807 (72.1%)
**Race (self-declared)**				
**American Indian/Alaska Native**	4 (0.428%)	10 (1.44%)	15 (1.71%)	29 (1.16%)
**Asian/Hawaiian/Pacific Islander**	7 (0.749%)	4 (0.576%)	8 (0.913%)	19 (0.758%)
**Black or African American**	13 (1.39%)	9 (1.29%)	16 (1.83%)	38 (1.52%)
**Multiple**	19 (2.03%)	65 (9.35%)	12 (1.37%)	96 (3.83%)
**Other**	5 (0.535%)	113 (16.3%)	0 (0%)	118 (4.71%)
**Unknown**	6 (0.642%)	20 (2.88%)	18 (2.05%)	44 (1.76%)
**White**	881 (94.2%)	474 (68.2%)	807 (92.1%)	2162 (86.3%)
**Ethnicity (self-declared)**				
**Hispanic/Latino**	18 (1.93%)	194 (27.9%)	167 (19.1%)	379 (15.1%)
**Not Hispanic/Latino**	910 (97.3%)	485 (69.8%)	701 (80%)	2096 (83.6%)
**Unknown**	7 (0.749%)	16 (2.3%)	8 (0.913%)	31 (1.24%)
**Age at enrollment (years)**				
**N-Missing**	0	191	0	191
**Mean (SD**^b^**)**	15 (2.19)	24.3 (3.54)	25.5 (4.46)	21 (6.07)
**Q1, Q3**	13; 17	22; 26	22; 27	16; 25
**Median**	15	23	24	21
**Age at last vaccination (years)**				
**N-Missing**	0	191	373	564
**Mean (SD**^b^**)**	8.43 (3.46)	20.4 (3.21)	25.8 (4.35)	16 (8.41)
**Q1, Q3**	5; 12	18; 21	23; 28	10; 22
**Median**	10	19	25	18
**Time from last vaccination to enrollment (years)**				
**N-Missing**	0	191	373	564
**Mean (SD**^b^**)**	6.58 (2.77)	3.38 (1.66)	0.012 (0.008)	4.05 (3.44)
**Q1, Q3**	4.65; 8.5	2.15; 4.01	0.005; 0.014	0.034; 6.43
**Median**	6.40	3.03	0.01	3.60

Q1, first quartile, Q3, third quartile.

^a^ Neutralizing antibody titer (mIU/mL), measured by the plaque reduction microneutralization assays (PRMN).

^b^ Standard Deviation.

### Measles-specific antibody assay

The methods described herein are similar or identical to those published for our previous studies [27, 28]. Neutralizing antibodies against measles virus (MV) were measured by a fluorescence-based plaque reduction microneutralization assay (PRMN) using a recombinant, GFP-expressing MV [27, 28]. The plates were scanned and counted on an automated Olympus IX71 Fluorescent microscope using the Image-Pro Plus Software Version 6.3 (Media Cybernetics; Rockville, MD). The 50% end-point titer (Neutralizing Doze, ND_50_) was calculated automatically using Karber’s formula, and transformed into mIU/mL (using the 3^rd^ WHO international measles antibody standard), as previously published [27, 28]. The variability of the PRMN assay in our laboratory, calculated as a coefficient of variation (CV) based on the log-transformed ND_50_ values of the third WHO standard, was 5.7% [28].

### HLA genotype assessment

For the Rochester and San Diego cohorts, HLA class I (A, B, and C) and class II (DRB1, DQA1, DQB1, DPA1, and DPB1) genotyping was performed by high-resolution PCR-SSP (Invitrogen; Carlsbad, CA), as previously described [29–31]. For the Rochester cohort, 249 subjects with European ancestry did not have HLA genotyping data (subjects were not genotyped for HLA); for these subjects, we imputed their HLA genotypes to four-digit allele codes (see below). For the U.S. cohort, HLA class I and class II allele genotyping was performed using high-throughput, high-fidelity genotyping with deep-sequencing technology that combines the advantage of long-range amplification with the power of high-throughput Illumina sequencing platforms that were developed at the Stanford Genome Technology Center (Stanford School of Medicine; Palo Alto, CA) [24, 32].

### Imputation of HLA

For the 249 subjects from the Rochester cohort without HLA data, we imputed their four-digit alleles at HLA loci A, B, C, DRB1, DQA1, DQB1, DPA1, and DPB1. Imputation was achieved with the SNP2HLA software using the Type-I Diabetes Genetics Consortium reference panel (n = 5,225) kindly provided by Jia, *et al* [33]. To perform imputation, we used all of the SNPs we had available for chromosome 6 in the HLA region based on a prior genome-wide association study (GWAS) [23, 25].

### Imputation of KIR types

The KIR genes are tandemly arrayed in a region of about 150 kb on chromosome 19q13.4. Because the genes have a high level of sequence similarity, the genes evolve by non-allelic homologous recombination, which explains the expansion and contraction of the KIR locus in terms of copy number [34]. We used the KIR*IMP software [35] to impute variables representing different definitions of KIR loci, including KIR gene copy number, KIR A or B haplotype, and gene-content haplotypes.

Twelve of the imputed genes are denoted: KIR2DS2, KIR2DL2, KIR2DL3, KIR2DP1, KIR2DL1, KIR3DP1, KIR2DL4, KIR3DS1, KIR2DL5, KIR2DS3, KIR2DS5, and KIR2DS1. The single digit after KIR corresponds to the immunoglobulin domain followed by the letter “D” for domain. A letter of L, S, or P is added to signify long or short cytoplasmic tail, or pseudogene, respectively. A final digit is added to distinguish between genes encoding the same protein structure. A thirteenth gene is denoted KIR3DL1ex4 or KIR3DL1ex9, which targeted copy number in exons 4 or 9, respectively, of the gene KIR3DL1. A 14th gene, KIR2DS4, was referred to as KIR2DS4DEL or KIR2DS4WT, corresponding to a 22-bp frameshift deletion or wild-type form, respectively. Another assay for this same gene detected both forms, and is denoted KIR2DS4TOTAL. In addition to these 14 genes, two types of haplotypes were imputed. The A/B haplotype denotes stable copy number (A haplotype), versus extensive copy number variation and many gene rearrangements. Different centromeric and telomeric motifs, which differ in the content and arrangement of genes, compose the KIR haplotypes. Further details of the KIR nomenclature and genomic structure are provided elsewhere [35].

To perform the imputation, we used prior GWAS SNPs to impute all SNPs genome-wide, then restricted to the imputed SNPs that had an imputation allele-dosage R^2^ of at least 0.8 (a measure of highly accurate imputation). Of the 301 SNPs that the KIR*IMP software used for imputation, we had 185 SNPs that were either measured or imputed well. The imputation accuracy was at least 90% for the majority of the KIR genes for each of the three cohorts, with imputation accuracy approximately 80% for the KIR haplotypes.

### Genetic ancestry and population stratification

Because the reference panels that are used to impute either HLA or KIR alleles contain only subjects with European ancestry, we restricted our analyses to those with European ancestry. We calculated European ancestry based on SNP data available from a prior GWAS. Genetic data was used to assign ancestry groups (African, European, or Asian) for individuals using the STRUCTURE software [26], and using the 1000 Genomes data as a reference. These estimates were done within cohort and platform (San Diego/550, San Diego/650, US/Omni 2.5, Rochester/Omni 1). Others [36] have shown that it is necessary to prune the SNPs used for eigenvectors to avoid having sample eigenvectors determined by clusters of SNPs at specific locations, such as the lactose intolerance gene, or polymorphic inversion regions. The SNPs used for STRUCTURE and eigenvectors were selected by LD pruning from an initial pool consisting of all autosomal SNPs with the following filters: SNPs with a minor allele frequency (MAF) < 5% were excluded; influential SNPs were removed (according to the following chromosome regions:chromosome 8 [bp 1–12700000], chromosome 2 [bp 129900001–136800000, 5700000–33500000], chromosome 4 [0900001–44900000]); and correlation (r^2^) pruning was used to subset to uncorrelated SNPs. These SNPs were input to the STRUCTURE program [26] to make ancestry “triangle” plots that depict the admixture proportions of ancestry groups for each subject. Subjects were classified into major ancestry groups based on the largest estimated STRUCTURE ancestry proportion.

Eigenvectors were estimated within ancestry groups and platform strata for refined control of population stratification. For this step, SNPs with a MAF < 0.01 were excluded, SNPs with a Hardy-Weinberg equilibrium (HWE) p-value < 0.001 were excluded, INDELS were removed, and pruning according to variance inflation factors was used. These data were then used with smartPCA to produce a set of eigenvectors using the normalization formulas of Price *et al*. [37], following the procedures of EIGENSTRAT. Tracy-Widom statistics were computed to potentially include eigenvectors to adjust for covariates if they had a p-value < 0.05.

## Statistical methods

To achieve the greatest power to detect HLA or KIR alleles associated with measles immune response phenotypes, we pooled our data across the three cohorts. To remove the effects of potential confounders so that the cohorts could be combined, we first screened for potential confounders relevant to each ancestry group and cohort. Possible confounders were screened for their association with the trait as follows. Any categorical variable with a very large number of categories was binned using hierarchical clustering on the estimated regression coefficients. All categorical variables were coded as dummy variables such that the most common category was used as baseline. Univariate linear models were then used to evaluate potential confounders. Variables that were marginally associated with the trait with a p-value < 0.1 were then included in backwards selection with a p-value threshold of 0.1. This somewhat liberal p-value threshold achieves the goal of controlling for potential confounding covariates. For the final association models, the covariates used for adjustment differed by cohort. For the San Diego cohort, we adjusted for subject year of birth, the operator that ran the assays, and having self-reported Asian, Hawaiian, or Pacific Islander ancestry. For the Rochester cohort, we adjusted for population stratification eigenvectors, self-reported Native American or Native Alaskan ancestry, operator, and assay run. For the US cohort, we adjusted for design factors (needing to repeat a sample, plate, and plate position), population stratification (eigenvectors and self-reported ancestry), type of vaccine, and subject age. Residuals from the adjusted model were used as the primary adjusted traits for analyses. HLA supertype variables were constructed for locus A and locus B using the amino acid sequences available from IPD such that HLA alleles within a given HLA locus that shared peptide binding specificity were grouped together [38, 39]. See S1 Table for grouping of HLA-B alleles into supertype categories.

The associations of HLA loci and alleles with a trait were evaluated by regression models by using the most frequent allele as a baseline, and all other alleles were coded according to the dose of the allele. Rare alleles (counts less than five) were grouped together into a rare-allele category. Four approaches were used to test associations: 1) a global test that simultaneously tested all alleles of a locus with the trait; 2) a global test that simultaneously tested all supertypes of a locus with the trait; 3) an allele test that tested the association of each allele within a locus with the trait; 4) a supertype test that tested the association of each supertype within a locus with the trait. The advantage of the global test is that it has more power to detect an effect if multiple alleles are associated, whereas the allele test is more powerful if only a single allele is associated. The tests of individual alleles or supertype categories were provided by the Wald statistic for each allele/supertype in the model. To control for multiple testing, we used the Bonferroni correction. For the HLA loci, there were eight global locus tests that required a p-value < 0.006 to achieve statistical significance; for KIR, there were 17 global tests that required a p-value < 0.003 for statistical significance. For the alleles tested, for the HLA loci, there were 249 allele-specific tests, resulting in a p-value < 0.0002 for statistical significance. We recognized that there might be some dependence among the tested alleles. Based on a method that considers the correlation among alleles within each locus [40], the estimated number of independent tests was the same as the actual number. For the KIR alleles and haplotypes, there were 27 allele/haplotype-specific tests, resulting in a p-value < 0.001 that is required for corrected statistical significance. Statistical analyses were performed with the R statistical software version 3.2.0 and PLINK [41].

## Results

### Characteristics of subjects

Our study cohort consisted of healthy children, adolescents, and adults between 11 and 41 years of age. Among 2,506 subjects enrolled into this study and retained in the analyses, because they had both antibody titer and genotype data available, 1,807 (72%) were male and 2,162 (86%) self-identified as Caucasian (Table 1). The mean neutralizing antibody titer for this cohort was 1,271 mIU/mL (SD; 1,652 mIU/mL). The median age at second (last) MMR vaccination was 18 years. There were no significant differences in neutralizing antibody titers between males and females in this cohort (p = 0.89).

### Associations of HLA genotypes with measles antibodies

**Fig 1:**
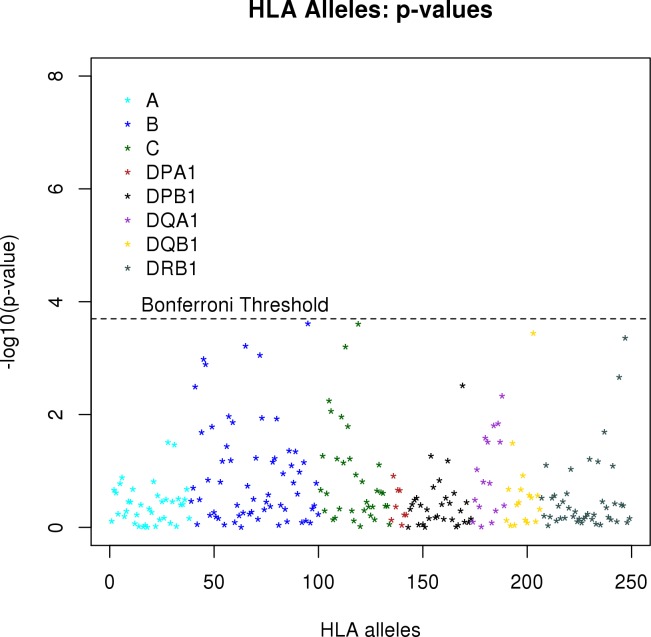
P-values for association of each HLA allele with neutralizing antibodies. The y-axis illustrates the–log_10_(p-value) for the association of an allele with neutralizing antibody titer based on linear regression of antibody titer on the dose of each allele. The Bonferroni threshold is based on 0.05/248.

**Fig 2:**
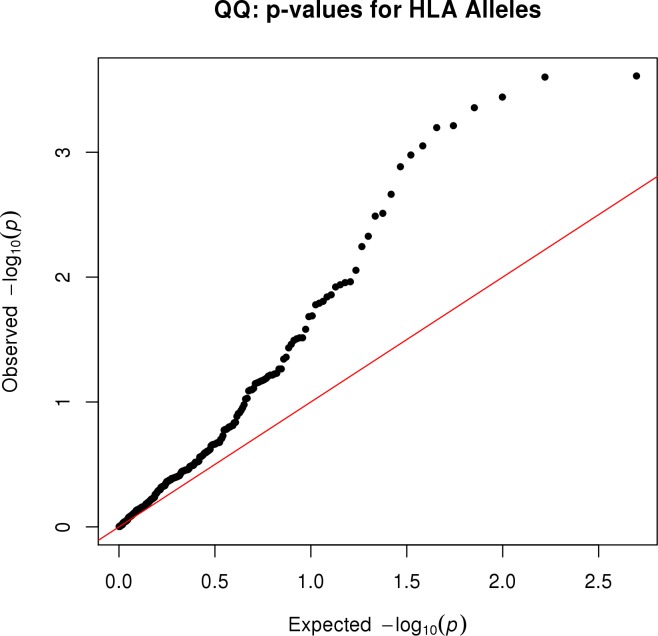
Quantile-quantile plot of p-values for HLA alleles associated with neutralizing antibodies. The p-values result from regression of neutralizing antibody titer on the dose of each allele, along with adjusting covariates that include eigenvectors to adjust for population stratification. The departure of the observed p-values from the diagonal line illustrates that many observed p-values departed from those expected when the null hypothesis of no association is true.

The association of each HLA allele with measles antibody titer is summarized in Fig 1, which depicts the–log_10_(p-value) for each of the alleles. This figure illustrates that the alleles from HLA loci B, DQB1, and DRB1 tend to have smaller p-values than alleles from the other loci. The quantile-quantile plot in Fig 2 shows that the p-values from all HLA alleles tend to be much more extreme than expected when there is no association of HLA alleles with measles antibody titers. Because we adjusted for potential population stratification, the departure of the p-values in Fig 2 from the diagonal line suggests that there are multiple HLA alleles associated with measles antibodies.

Table 2 shows outcomes for associations between HLA alleles with measles antibody titers across study cohorts, with presented results restricted to those alleles that had p-values ≤ 0.001. The global tests (threshold p-value < 0.0002) did not detect significant associations of HLA loci with antibody titers. For the HLA-B locus, we found four alleles with p-value < 0.001: B*07:02, B*35:17, B*40:01, and B*57:01. The effects of these alleles are represented by the regression coefficients from a regression model with the most frequent allele at a locus serving as a baseline, and all other alleles coded according to their dose in a genotype. A negative coefficient indicates that an allele is associated with a lower level of neutralizing antibody than the baseline allele, and a positive coefficient indicates the opposite effect. For each of these four alleles, the direction of the effects were consistent across all three cohorts, with each of the four alleles associated with a lower level of neutralizing antibody compared to the baseline allele: B*07:02.

**Table 2:** HLA allelic associations with measles-specific neutralizing antibody titers in the pooled data of 2,506 subjects and the three separate cohorts: Rochester, San Diego, and US. Results are presented for alleles that have p-values < 0.001 for association with neutralizing antibody titers.

HLA Locus	Allele Group	Regression Coefficient^a^	Number of Carriers	Mean (mIU/mL)	Median (mIU/mL)	Lower Quartile (mIU/mL)	Upper Quartile (mIU/mL)	Allele P-value	Global P-value
Overall			2,506	1,271	803	383	1,607		
HLA-B			2,453	1,264	799	378	1,583		0.052
	B*07:02 (baseline) Pooled	baseline	589	1,457	1,005	473	1,851	baseline	
	Rochester	baseline	248	1,355	990	471	1,852	baseline	
	San Diego	baseline	149	1,582	1,119	538	2,114	baseline	
	US	baseline	192	1,494	885	409	1,632	baseline	
	B*35:17 Pooled	-0.96	12	443	243	111	622	0.0006	
	Rochester	NA	---	---	---	---	---	---	
	San Diego	-0.83	6	611	476	243	781		
	US	-1.27	6	274	177	106	244		
	B*40:01 Pooled	-0.23	263	1,126	689	365	1,549	0.0009	
	Rochester	-0.11	117	1,158	708	436	1,700		
	San Diego	-0.43	70	1,033	716	306	1,411		
	US	-0.18	76	1,62	632	362	1,476		
	B*57:01 Pooled	-0.32	155	1,040	649	298	1,280	0.0002	
	Rochester	-0.29	52	1,033	748	340	1,383		
	San Diego	-0.37	40	1,319	693	373	1,161		
	US	-0.31	63	867	433	258	1,205		
HLA-C			2,437	1,257	799	378	1,583		0.282
	C*07:02 (baseline) Pooled	baseline	647	1,440	944	434	1,820	baseline	
	Rochester	baseline	270	1,367	997	476	1,887	baseline	
	San Diego	baseline	184	1,402	1,007	446	1,822	baseline	
	US	baseline	193	1,577	835	379	1,622	baseline	
	C*06:02 Pooled	-0.20	395	1,082	660	328	1,342	0.0006	
	Rochester	-0.16	129	1,084	717	348	1,493		
	San Diego	-0.18	102	1,293	820	410	1,514		
	US	-0.23	164	951	546	273	1,202		
	C*08:02 Pooled	-0.31	151	1,197	631	303	1,096	0.0003	
	Rochester	-0.21	40	966	859	399	1,321		
	San Diego	-0.58	49	917	625	257	992		
	US	-0.22	62	1,567	605	308	1,055		
HLA-DQA1			2,454	1,264	799	377	1,583		0.230
	DQA1*01:02 (baseline) Pooled	baseline	825	1,337	919	425	1,721	baseline	
	Rochester	baseline	318	1,359	998	473	1,771	baseline	
	San Diego	baseline	230	1,390	990	411	1,829	baseline	
	US	baseline	277	1,269	833	397	1,488	baseline	
	DQA1*05:05 Pooled	-0.14	477	1,190	772	350	1,444	0.0047	
	Rochester	-0.08	127	1,479	1,057	475	1,891		
	San Diego	-0.16	179	1,160	760	388	1,276		
	US	-0.16	171	1,005	594	299	1,268		
HLA-DQB1			2,447	1,264	797	377	1,583		0.011
	DQB1*03:01 (baseline) Pooled	baseline	803	1,291	804	354	1,532	baseline	
	Rochester	baseline	267	1,399	998	478	1,834	baseline	
	San Diego	baseline	261	1,169	720	343	1,334	baseline	
	US	baseline	275	1,303	649	304	1,388	baseline	
	DQB1*06:02 Pooled	0.17	551	1,349	968	471	1,754	0.0004	
	Rochester	0.11	225	1,387	997	475	1,772		
	San Diego	0.29	141	1,431	1,030	485	1,862		
	US	0.16	185	1,239	906	437	1,583		
HLA- DRB1			2,454	1,264	799	377	1,583		0.046
	DRB1*07:01 (baseline) Pooled	baseline	585	1,130	732	357	1,470	baseline	
	Rochester	baseline	206	1,104	752	361	1,487	baseline	
	San Diego	baseline	154	1,200	731	405	1,529	baseline	
	US	baseline	225	1,107	730	345	1,417	baseline	
	DRB1*15:05 Pooled	0.99	12	2,547	2,235	1,244	3,513	0.0004	
	Rochester	NA	---	---	---	---	---	---	
	San Diego	0.94	12	2,547	2,235	1,244	3,513		
	US	NA	---	---	---	---	---	---	

^a^ Regression coefficients from regression model with the most frequent allele at a locus serving as a baseline, and all other alleles coded according to their dose in a genotype. A negative coefficient indicates that an allele is associated with a lower level of neutralizing antibody than is the baseline allele, and a positive coefficient indicates the opposite effect.

The HLA-C locus had two alleles, C*06:02 and C*08:02, with p-value < 0.001; both of these alleles had lower antibody levels compared to the baseline allele, C*07:02. Because there is strong linkage disequilibrium among the alleles from the HLA-B and HLA-C loci, we evaluated the joint effects of B alleles and C alleles, adjusted for each other. We focused on the B alleles that had small p-values (B*35:17, B*40:01, and B*57:01), and C alleles that also had small p-values (C*06:02 and C*08:02). The results showed that the B alleles retained small p-values (all p-values <0.006), while the effects of the C alleles dramatically diminished (p-values > 0.3), suggesting that the effects of the C alleles might be driven primarily by their associations with the B alleles.

At the HLA-DQA1 locus, the allele DQA1*05:05 had a lower antibody level than the baseline allele DQA1*01:02. At the HLA-DQB1 locus, the allele DQB1*06:02 had a higher level of antibody compared to the baseline allele DQB1*03:01. Finally, at the HLA-DRB1 locus, the low frequency allele DRB1*15:05 had a higher antibody level than the baseline allele DRB1*07:01. These suggestive associations must be carefully considered due to the absence of significant global tests.

There was no evidence of HLA-A supertype association with neutralizing antibody titers (global p-value, 0.99; allele p-value range of 0.76 to 0.93) (data not shown). In contrast, there were suggestive associations between neutralizing antibody titers and the HLA-B supertype at both the global (p = 0.003) and the individual supertype levels. The alleles contributing to each supertype category at locus B can be found in S1 Table. We observed an increase in neutralizing antibody titer for HLA B7 supertype (baseline) relative to all other supertypes, while all other HLA-B supertype categories were associated with a lower neutralizing antibody titer. This is consistent with the allelic-level trends. The p-values for the category-level associations are given in **S2** Table. In particular, B44 (1,204 mIU/mL; p = 0.0085) and B58 (1,207 mIU/mL; p = 0.0018) supertypes demonstrated the strongest associations with lower mean titers of measles antibodies.

### Associations of KIR genotypes with measles antibodies

**Fig 3:**
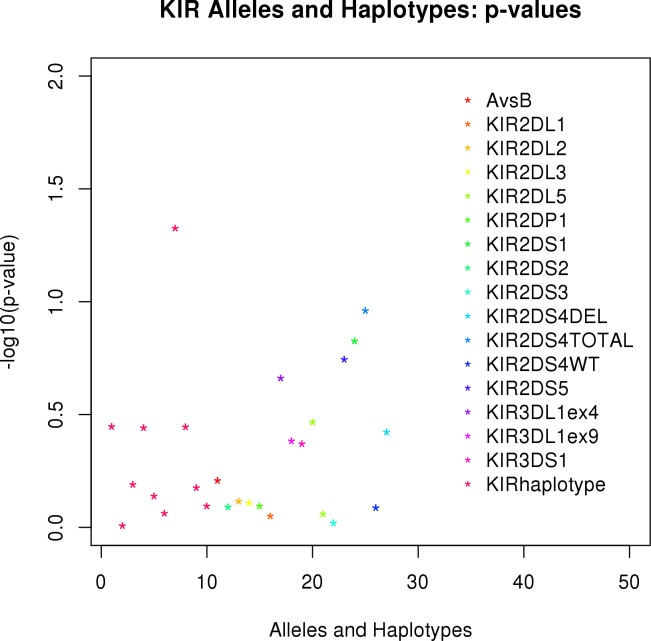
P-values for association of alleles and haplotypes for KIR loci with neutralizing antibodies. The y-axis illustrates the–log_10_(p-value) for the association of an allele or haplotype with neutralizing antibody titer based on linear regression of antibody titer on the dose of each allele or haplotypes. The Bonferroni threshold is based on 0.05/27.

**Fig 4:**
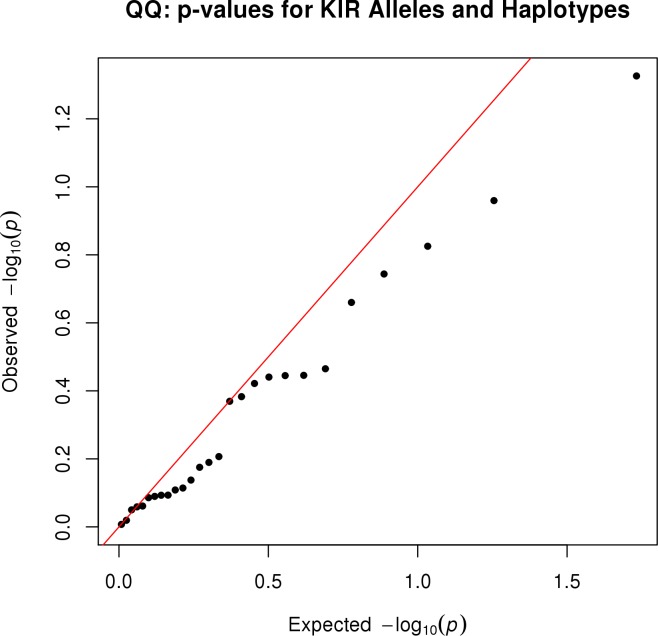
Quantile-quantile plot of p-values for KIR alleles and haplotypes associated with neutralizing antibodies. The p-values result from regression of neutralizing antibody titer on the dose of each allele or haplotype, along with adjusting covariates that include eigenvectors to adjust for population stratification. The observed p-values were close to the diagonal line, suggesting that the observed p-values had a distribution that would be expected when the null hypothesis of no association is true.

The association of each KIR allele and haplotype with measles antibody titers is summarized in Fig 3. This figure illustrates that the KIR alleles and haplotypes show no evidence of association with measles antibody titer. This is further illustrated in Fig 4, which shows that the p-values are generally close to (or below) the diagonal in the quantile-quantile plot. We interpret this to mean that none of the KIR alleles and haplotypes are associated with measles antibody titers. This quantile-quantile plot also shows that we have controlled for population stratification, as upward departure from the diagonal would be expected if we had not adequately controlled for population stratification. More detailed results on the associations between KIR genotypes/haplotypes and measles-specific antibody titers are presented in Table 3. Because the effects of KIR loci were dramatically negative (smallest p-value over all 17 tested KIR loci was 0.13), we chose not to explore combinations of HLA and KIR alleles.

**Table 3:** KIR genotype associations with measles vaccine-specific neutralizing antibody titers.

KIR genes	P-value
KIR Haplotype	0.71
A vs B	0.64
KIR2DS2	0.82
KIR2DL2	0.78
KIR2DL3	0.79
KIR2DP1	0.82
KIR2DL1	0.90
KIR3DP1	monomorphic^*^
KIR2DL4	monomorphic^*^
KIR3DL1ex4	0.24
KIR3DL1ex9	0.44
KIR3DS1	0.45
KIR2DL5	0.65
KIR2DS3	0.95
KIR2DS5	0.20
KIR2DS1	0.17
KIR2DS4TOTAL	0.13
KIR2DS4WT	0.83
KIR2DS4DEL	0.40

*Monomorphic loci had no genetic variation so associations could not be tested.

## Discussion

We have previously described associations of HLA alleles with inter-individual variation in humoral immune response to measles vaccine. To expand upon our prior work, we performed a pooled analysis—with a much larger sample size—of the association of HLA loci with vaccine-induced immune measures using data obtained from three separate cohorts in order to maximize power. In addition, we examined HLA in terms of peptide-binding specificities through the use of HLA supertypes. Finally, we examined whether variation within the KIR genes, which display extensive genetic diversity, influences measles vaccine-induced antibody responses.

In this large cohort, we demonstrated suggestive associations between several allelic variants of the HLA-B, -DQB1, and -DRB1 loci and neutralizing antibody titers. HLA-B (*57:01), HLA-DQB1 (*06:02), and DRB1 (*15:05) alleles were associated with variation in antibodies, even after adjusting for relevant covariates. This study also showed that HLA-B supertypes (B44, B58) were marginally associated with measles antibodies. Although the associations of antibody titers with individual HLA loci or alleles did not achieve statistical significance according to the strict Bonferroni criterion, there is suggestive evidence that multiple alleles at the HLA loci are associated with neutralizing antibody titers, as illustrated by the excessive number of small p-values in the quantile-quantile plot. Despite our large study, the power to detect associations was limited by several factors. First, the small allele frequencies of some of the HLA alleles limit power. Second, global tests of an HLA locus can have reduced power when there are many alleles, of which only a small subset of alleles are associated with antibody titers.

In contrast to the multiple HLA alleles associated with response to measles vaccination, we were unable to find any evidence for associations of KIR loci or haplotypes with measles antibody titers. In this regard, it is worth noting that some KIR alleles are not expressed on the surface (NK cells and T lymphocytes), may not play a role in B cell development and antibody production, and/or may not be functional. Alternative approaches, including studies of functionally relevant KIR/HLA pairs based on the KIR/HLA allele frequencies, might be needed to understand the relationship between KIR alleles and their HLA ligand genes [42].

HLA associations found in this large combined cohort (n = 2,506) were compared to the ones previously found in a dataset of 346 Rochester cohort subjects (this Rochester cohort is part of the combined cohort of 2,506) [8]. Finding B*57:01 and DQB1*06:02 allele associations in both the smaller (n = 346) and combined (n = 2,506) cohorts could be indicative that these alleles (B*57:01 and DQB1*06:02) are associated with the neutralizing antibody phenotype. Also, the DRB1*15 allele appears to be in linkage disequilibrium (LD) with the HLA-DQB1*06:02 allele. Similarly, we found suggestive associations between several alleles from the HLA-B locus that belong to the major HLA-B supertypes (alleles with overlapping peptide-binding specificities), such as B44 (*40:01) and B58 (*57:01), which were previously found in a small dataset of Rochester cohort subjects (n = 346) that is part of this large combined cohort [43]. The nature of the association between HLA class I alleles and neutralizing antibody responses is not clear, as CD8+ T cell activity is not usually associated with humoral immunity. It can be proposed that antigen-presenting cells may present exogenous MV overlapping epitopes via cross-presentation by both class I and class II HLA molecules, as was recently described for the exogenous HIV-1 antigen [44]. By identifying genetically restricted MV-derived peptides, it may be possible to develop new multi-peptide HLA supertype vaccines administered with specific adjuvants [45, 46]. Studies have also shown that CD8+ T cell responses correlate with viral RNA levels in MV-infected macaques [47]. One possible explanation is that T cell responses facilitate viral clearance and thereby alter the availability of MV antigen for surface immunoglobulin on B cells. This may indirectly affect B cell activation and antibody production.

The strength of this work is that we have improved upon the rigor of our prior studies. In this current work, we utilized model selection to select the most relevant covariates to adjust for while evaluating the role of HLA or KIR alleles. We have also carefully controlled for the effects of population stratification by restricting to subjects with European ancestry by using a combination of the STRUCTURE race categorization and self-declared race, and by adjusting for population stratification in the analyses using eigenvectors estimated from genome-wide SNP measurements from a prior GWAS. We comprehensively examined the association of KIR and HLA genes on MV-induced immunity via state-of-the-art imputation methods. No previous measles vaccine studies have utilized an imputation approach to understand MV immunity. We pooled all of our available data across well-characterized and independent cohorts to gain power to detect associations. Additionally, we utilized an improved high-throughput uniform assay to reliably quantify neutralizing antibody titers. Although this assay does not measure all antibodies that may influence viral clearance and protection (i.e., antibody-dependent cellular cytotoxicity/ADCC antibodies) [48], it is the currently accepted "gold standard" assay in measles serology and provides measures (neutralizing antibody titers) used as correlates of protection against measles [28, 49]. Finally, we have been more conservative in our thresholds for statistical significance and acknowledged the issue of multiple testing.

While our study has many strengths, limitations are also present. We had insufficient numbers of other racial groups to conduct an independent study of response. Further studies are required to examine KIR/HLA allelic associations with measles vaccine immune response phenotypes in cohorts of different ancestry. Greater sample size and an increased sampling of other races are advisable to resolve these problems. Numerous statistical tests were carried out for this study; therefore, the probability of false-positive results is present. Also, the association between specific HLA alleles, such as B*35:17 and DRB1*15:05, must be interpreted with extreme caution due to the low frequency of these alleles. The low frequency of these alleles may imply that this association is unlikely to be informative in understanding the mechanisms of measles vaccine-induced antibody response. Even so, many of the associations discussed were observed in our previous measles vaccine studies with smaller subsets of these cohorts and less rigorous techniques, and our results make sense biologically in the context of what is known for the role of HLA in the vaccine-induced immunity framework. This lends confidence to our findings [8, 43].

In conclusion, our results demonstrate that alleles at the HLA-B (*57:01), -DQB1 (*06:02), and -DRB1 (*15:05) loci show some level of association with neutralizing antibody response, albeit at levels that do not achieve statistical significance. Based on the totality of all tested HLA alleles, there is evidence that the effects of HLA alleles on neutralizing antibody response are not simple chance occurrences, but rather a large number of alleles having small to modest effect sizes. Future studies will determine the direct effects and downstream functional consequences on neutralizing antibody phenotype of HLA (and other gene polymorphisms) that we have identified in the combined Rochester, San Diego, and U.S. cohorts. Knowledge about the immunogenetic impact of gene polymorphisms promises to lead to an enhanced mechanistic understanding of the immune response to MV, generate general principles and insights into the immunogenicity of other live viral vaccines, and inform the directed development of vaccine candidates against measles and other viruses.

## Supporting information

S1 Table The HLA alleles contributing to each supertype category at the B locus. (DOCX)

S2 Table HLA B supertype associations with measles-specific neutralizing antibody titers. (DOCX)
