# Supplementary material for: A large population-based association study between HLA and KIR genotypes and measles vaccine antibody responses
Source: PLoS One. 2017 Feb 3;12(2):e0171261. doi: 10.1371/journal.pone.0171261 (PMC5291460; doi:10.1371/journal.pone.0171261)
Supplement: S1 Table — (DOCX) [file pone.0171261.s001.docx]

**S1 Table.** The HLA alleles contributing to each supertype category at the B locus.

| HLA-B Supertypes | Alleles Included in HLA Supertype Category |
| --- | --- |
| 0 (Alleles not found in a supertype) | 40:02, 41:02, 18:01, 08:01, 39:06, 35:08, 44:05, 47:01, 42:01, 35:49, 15:15, 07:06, 35:10, 57:03, 44:27, 15:04, 14:03, 15:30, 35:12, 42:02, 39:05, 39:10, 35:11, 44:07, 15:35, 35:40,35:17, 15:48, 39:11, 15:24, 39:08, 40:16, 40:03, 38:05, 40:04, 27:14, 40:27, 07:44, 15:37, 35:05, 15:11, 08:12, 07:25, 15:72, 45:04, 50:02, 07:07, 15:47, 40:08, 15:07, 35:43, 18:03, 15:22, 15:39, 40:05, 15:25, 35:67, 15:27, 3915, 35:04, 44:04, 18:13, 07:14, 14:06, 50:04, 81:01, 18:02, 78:05, 35:23, 27:13, 39:24, 40:12, 38:04, 44:37, 40:10, 35:14, 59:01, 44:06, 42:05, 27:12, 08:18, 08:09, 39:09, 41:03, 82:01, 78:02, 35:42, 48:03 |
| B7 (Model baseline) | 07:02, 07:03, 07:04, 07:05, 15:08, 35:01, 35:02, 35:03, 51*, 53:01, 54:01, 55:01, 55:02, 56:01, 56:02, 67:01, 78:01 |
| B27 | 14:01, 14:02, 15:03, 15:09, 15:10, 15:18, 27:01, 27:02, 27:03, 2704, 2705, 2706, 2707, 27:08, 38:01, 38:02, 39:01, 39:02, 39:03, 39:04, 48:01, 48:02, 73:01 |
| B44 | 37:01, 40:01, 40:06, 41:01, 44:02, 44:03, 45:01, 49:01, 50:01 |
| B58 | 15:16, 15:17, 57:01, 57:02, 58:01, 58:02, 58:03, 58:04, 58:05, 58:06 |
| B62 | 13:01, 13:02, 15:01, 15:02, 15:06, 15:12, 15:13, 15:14, 15:19, 15:21, 46:01, 52* |

*2-digit allele supertype.
