# Supplementary material for: A large population-based association study between HLA and KIR genotypes and measles vaccine antibody responses
Source: PLoS One. 2017 Feb 3;12(2):e0171261. doi: 10.1371/journal.pone.0171261 (PMC5291460; doi:10.1371/journal.pone.0171261)
Supplement: S2 Table — (DOCX) [file pone.0171261.s002.docx]

**Table S2.** HLA allelic associations with measles-specific neutralizing antibody titers in the combined cohort of 2,506 subjects (Rochester, San Diego, and US).

| **HLA Locus** | **Allele** | **Number**  **of Carriers** | **Mean**  **(mIU/mL)** | **Median (mIU/mL)** | **Lower Quartile (mIU/mL)** | **Upper Quartile (mIU/mL)** | **Allele**  **P-value** | **Global**  **P-value** |
| --- | --- | --- | --- | --- | --- | --- | --- | --- |
| Overall |  | 2,506 | 1,271 | 803 | 383 | 1,607 |  |  |
| HLA Supertype |  |  |  |  |  |  |  |  |
| Class IB |  | 2,453 | 1,264 | 799 | 378 | 1,583 |  | 0.0031 |
| Baseline | B7 | 1,216 | 1,393 | 881 | 414 | 1,788 |  |  |
| Alleles not found in a supertype | 0 | 955 | 1,178 | 759 | 363 | 1,506 | 0.0037 |  |
|  | B27 | 548 | 1,210 | 728 | 373 | 1,423 | 0.0110 |  |
|  | B44 | 1,024 | 1,204 | 782 | 360 | 1,498 | 0.0085 |  |
|  | B58 | 215 | 1,207 | 678 | 298 | 1,286 | 0.0018 |  |

P-values from linear models with most frequent allele set to baseline. Associations were adjusted for cohort and cohort-specific confounders. Global p-values test the simultaneous association of any alleles of an HLA locus with antibody titer. Allele-level p-values test the effect of a given allele while adjusting for other alleles and covariates. Allele-level p-values less than 0.05 and all global p-values are shown.
